# Paul Workman: Drugging the Cancer Genome

**DOI:** 10.1016/j.trecan.2016.10.004

**Published:** 2016-10

**Figure:**
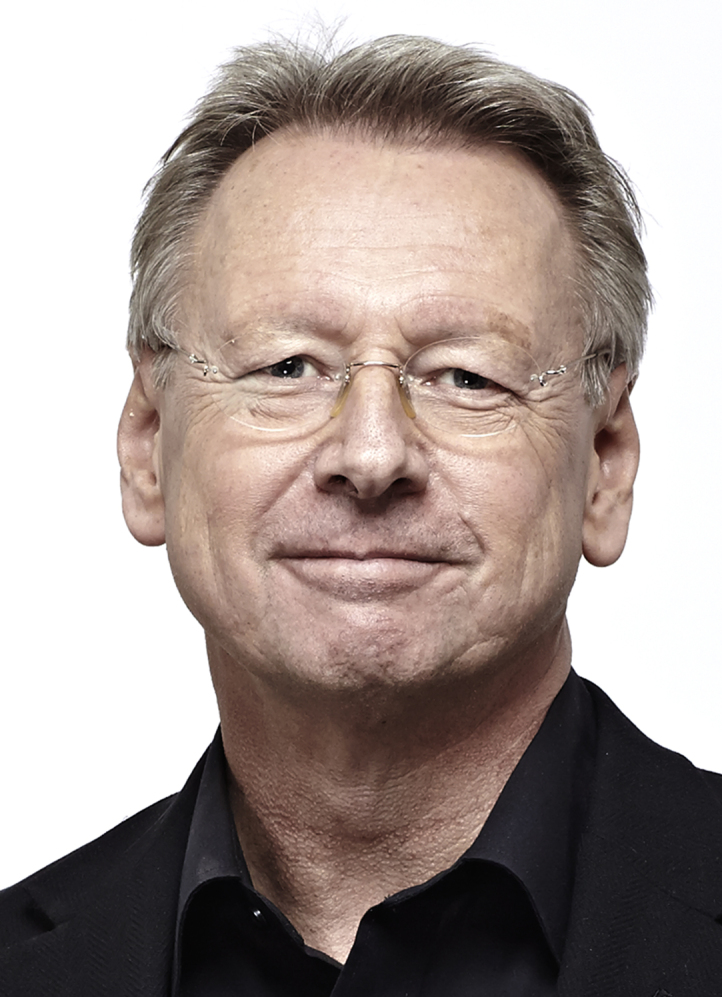

Fast-paced advances in cancer biology, genomics, and technology have not always translated rapidly into the swift development of new cancer therapies. Changing the landscape of cancer drug discovery is Paul Workman, CEO and President of The Institute of Cancer Research (ICR, London) and Leader of the Signal Transduction and Molecular Pharmacology Team in the Cancer Research UK Cancer Therapeutics Unit at ICR. Dr Workman spearheads the largest academic drug discovery multidisciplinary center, with an unmatched track record in bringing innovative drug candidates to clinical trials and to patients. Here, he reflects on the challenges and multiple opportunities ahead and shares his vision guiding the next decades of cancer drug research and development.

## How has cancer drug discovery changed over the past decades?

Looking back on this history is incredibly interesting and informative. I think the most dramatic difference over the past five or six decades, much of which I have lived through during my own research career, has been the transition from the initial period focusing on cytotoxic chemotherapy to the modern era of molecularly targeted drugs. Both periods led to major improvements in outcomes for patients with cancer. The chemotherapy era began during the 1940s and 1950s with the introduction of drugs that damaged DNA, often by crosslinking of the Watson-Crick double helix. The initial clinical success, which saw regressions in lymphoid tumors with nitrogen mustard (originating from chemical warfare work) introduced in 1942, led to the discovery and development of chemically less reactive and better tolerated drugs. These included ICR drugs, such as melphalan, chlorambucil, and busulphan, as well as carboplatin, which followed on from cisplatin (approved for ovarian cancer in 1978); all of these drugs also act as DNA-damaging agents and crosslinkers, and each is still in clinical use.

Another class of cytotoxic agents that showed early promise is the so-called ‘antimetabolites’, which work by blocking the enzymes involved in the synthesis of DNA from its chemical building blocks. These include drugs such as methotrexate and 5-fluoruracil, which again are still widely used. Likewise, many natural products were identified that block cell division, for example by binding to tubulin, including vincristine, vinblastine, and later paclitaxel (Taxol^®^). In fact, Bristol-Myers Squib's drug paclitaxel, which was isolated from the bark of the Pacific yew tree during the1960s and approved in 1992, was the first to be described as a billion-dollar ‘blockbuster’ oncology drug. Natural product topoisomerase inhibitors exemplified by irinotecan also came through to the clinic. These and other cytotoxic agents were in some cases rationally designed to act on the drug target, as with antimetabolites, or alternatively often were identified by screening for agents that inhibit cancer cell division and kill cancer cells, initially regardless of mechanism, as in the case of the natural products.

Following significant success with alkylating agents and methotrexate, for example, as single agents in patients with cancer, the first really big breakthroughs in the clinic came from the use during the 1960s of the combination of cytotoxic chemotherapy drugs from different classes to create a revolutionary curative regimen for acute lymphoblastic leukemia in children and then in Hodgkin's lymphoma and later non-Hodgkin's lymphoma, both in adult patients. The main idea was that, through the combination of mainly cytotoxic drugs having distinct cellular mechanisms of action and nonoverlapping adverse effects, one could obtain greater antitumor benefit while avoiding lethal toxicity to the patient. This was important because the cytotoxic chemotherapy agents not only kill proliferating cancer cells, but also destroy rapidly dividing normal cells, such as in the gut and bone marrow. The increased anticancer effectiveness of combination chemo, and the concept that these cocktails could reduce the occurrence of drug resistance with single agents, was supported by experience of using combination therapy to successfully treat infectious diseases, such as tuberculosis, and later HIV.

Much of the clinical progress was underpinned by lab research in mouse leukemia models and valuable concepts such as scheduling and maximizing fractional cell kill. After the early success in leukemias and lymphomas, the continued use of cytotoxic drug combinations also began to impact treatment of more common solid tumors, such as breast and colorectal cancer. This fascinating era of cytotoxic chemotherapy is well described in Vincent DeVita's recent book *The Death of Cancer*, which also covers the personalities and politics involved in US President Nixon's so-called ‘War on Cancer’ during the 1970s. I wrote a three-part essay about all this in my blogi.

Although still widely used and valuable clinically, the big limitation of the cytotoxic chemo drugs is their killing of all rapidly dividing cells, giving rise to severe adverse effects and, hence, a narrow therapeutic window. Also, there is the problem of drug resistance, even with a combination of cytotoxic drugs in cocktails. Furthermore, these are usually one-size-fits all treatments with little or no ability to predict which particular patients will respond.

Just as there was a realization that cytotoxic drugs had reached a plateau in their effectiveness, the next real clinical breakthrough came from the introduction of targeted therapies. These new therapies were based on our growing understanding of the causal molecular mechanisms underpinning cancer and very much exploited the extraordinary discoveries of the many genes and signaling pathways that are hijacked by cancer cells. That knowledge has come from basic, fundamental, blue-skies research that in fact benefited greatly from the increased funding allocated to the War on Cancer since 1971. The period during the 1970s and 1980s was the initial heyday for the discovery of oncogenes and tumor suppressor genes.

## What do you see as the main milestones and challenges for impactful personalized drug discovery?

There was in fact a frustrating 20-year gap before academic researchers and the pharmaceutical industry converted our growing knowledge of the fundamental basis of cancer into the exciting new wave of molecularly targeted drugs, starting with the approval in 1998 of trastuzumab (Herceptin^®^), a monoclonal antibody that binds to the extracellular domain of the oncogenic HER2 receptor tyrosine kinase and used to treat HER2-positive metastatic breast cancer. Next, in 2001, Novartis's BCR-ABL kinase tyrosine kinase inhibitor imatinib (Glivec) was approved for BCR-ABL-positive chronic myeloid leukemia. Here, we had a small-molecule drug that specifically blocked the single, translocated, activated oncogene product that drives the disease and totally transformed the outcome for patients. It was followed by the small molecules gefitinib (Iressa^®^), which I was involved in while at AstraZeneca, and also erlotinib (Tarceva^®^), both of which inhibit the epidermal growth factor receptor (EFGR) tyrosine kinase. An important breakthrough with these drugs was the discovery in 2004 that it was patients with non-small lung cancer whose tumors harbored oncogenic activating mutations in EGFR who responded to them. Meanwhile, cetuximab (Erbitux^®^), a monoclonal antibody that acts on the extracellular domain of EGFR, was approved for combination therapy in colon cancer in 2004. It was later shown only to be active in patients with KRAS wildtype cancers. The first clinically proven antiangiogenic drug bevacizumab (Avastin^®^) is another antibody, in this case to vascular endothelial growth factor (VEGF), which was approved for colon cancer in 2004.

This was an incredibly exciting time. Progress was accelerated not only by the increased fundamental knowledge about the molecular basis of what we now call the ‘Hallmarks of Cancer’, and the eventual shift in therapeutic mindset that I mentioned above, but also by technological advances, such as high-throughput screening and structure-based drug design for small-molecule inhibitors as well as recombinant DNA technology for biological agents. The new wave of biotechnology companies, such as Sugen, whose researchers I worked closely with and also many others, such as Amgen and Genentech, had a big impact. Improved animal models had an important role, although these are still by no means perfect, progressing from mouse leukemias as the main screening model through human tumor cell line panels (latterly molecularly characterized) and conventional human tumor xenografts and then genetically engineered mouse models all the way to the current favorites, which are organoid cultures and more freshly produced patient-derived xenografts (PDXs). In retrospect, I think the 20-year delay between the discovery of SRC and other oncogenic kinases and the approval of the first kinase inhibitor drug was caused by the need to switch the collective mindset of the oncology field, especially but not solely in industry, away from the cytotoxic paradigm to what we now variously call stratified, personalized, or precision molecular medicine. Notably of course, it was bridged by the antiestrogens, initially tamoxifen (a failed contraceptive), for the treatment of estrogen receptor-positive breast cancer, which was in fact initially approved in the UK as early as 1972, despite the focus on cytotoxic chemo at that time.

## How are patients with cancer benefiting from ‘drugging the genome’ approach?

I think I am responsible for coining this term ‘drugging the cancer genome’ and promulgating it in my talks and reviews! I find it a useful term conceptually to describe the new genome-centric approach that has dominated the cancer drug discovery horizon from 2000 to now. What we have seen over this period is a dramatic increase in the pace and scale at which cancer genes have been discovered, most especially as a result of the revolution in high-throughput genome sequencing. This was first exemplified in the UK Cancer Genome Project's identification of mutant BRAF as a major oncogene in melanoma, which led rapidly to the discovery and approval of BRAF and MEK inhibitors for that disease. Further rapid progress has been made by international efforts, such as the The Cancer Genome Atlas consortium.

In parallel, also important has been the co-development of companion biomarkers for patient selection, which is crucial for the implementation of personalized medicine. At the simplest level, this involved having a companion biomarker assay for a single predictive marker, for example, a BRAF mutation or HER2 amplification. Beyond that, many patients now benefit from sequencing large numbers of cancer genes through gene panel tests and, in many instances, whole-exome or entire genome sequencing as well as gene expression profiling, with treatment decisions being made based on this. This has for example been valuable in identifying mechanisms underlying exceptional responders or resistance to targeted therapies. I hope that we are moving towards routine gene panel and whole-exome/genome sequencing to guide therapy in a routine setting.

Beyond predictive assays, target engagement biomarkers and additional biomarkers of downstream biochemical and biological effects have been critical in informing go/no go decision-making and the selection of dose and schedule of administration for molecularly targeted agents. I have codified this within a conceptual and practical framework that I christened the ‘Pharmacological Audit Trail’, which has proved valuable.

Drugging the cancer genome has progressed beyond the now common development of drugs that block the function of driver oncogenes to include the approach of synthetic lethality, as exemplified by the approval in 2014 of olaparib for patients with ovarian cancer with BRCA mutations, and its exciting potential in patients with prostate cancer with BRCA and other DNA repair defects.

However, there is still a long way to go in drugging the cancer genome before we can say that we are even approaching genome-scale coverage. We need to continue to expand the number of cancer gene defects for which we have corresponding drugs. Although there are around 500 cancer-causing proteins, for example as listed in the Catalogue of Somatic Mutations in Cancer (COSMIC), we only have drugs for about 5% of these. We need to focus more effort on discovering innovative drugs that act on currently untargeted cancer proteins, as opposed to developing ‘me too’ drugs that simply reproduce the effects of existing medicines. Many cancer genes are technically hard to drug because of the absence of readily druggable pockets or protein–protein interactions, but the envelope of druggability is being greatly extended, as shown by the inhibitors of the BCL2 family. In general, tumor suppressor genes are tough to drug but synthetic lethal opportunities are now available. Standout examples of cancer genes that are not yet drugged are *RAS*, *MYC* and *P53*. We have to get drugs for all the key cancer genes if we are to approach the aspirational goal of routine genome sequencing for all patients with cancer and linking this to the choice of personalized single drugs or combinations for all cancer genome states.

## What are the principles behind running a large successful academic drug discovery team?

I think I can best answer this by first speaking from my own personal experience of building and running such a team and then broadening to more general observations.

I came to ICR in 1997 to be Director of the Cancer Research UK (CRUK) Therapeutics Unit. I had previously spent around 20 years in academia at the University of Leeds, the MRC Oncology Unit at Cambridge, a brief sabbatical at Stanford and SRI International, and then in Glasgow as Director of Laboratory Research in Medical Oncology at the Beatson Laboratory.

Rather unusually for that time, from 1993 to 1997, I then spent 4 years in a senior leadership position at AstraZeneca (then called Zeneca) discovering drugs acting on new molecular targets, including gefitinib, and also initiating and running the strategic alliance on kinase inhibitors with Sugen. Throughout all this time, my focus had been on new drug discovery and development: straddling the end of the cytotoxic era, targeting tumor hypoxia, discovering early kinase inhibitors and other signaling inhibitors, and then running a wide range of projects in industry.

I came to the conclusion that something was missing in the ecosystem. Industry tended to be rather risk averse and reluctant to take on early-stage projects where proof of concept for novel targets was lacking. There was also increasing pressure to consider commercial aspects and blockbuster potential at too early a stage. Academia was reasonably comfortable with early risk but lacked resources, firepower, professional project leadership, and especially medicinal chemistry skills, multidisciplinary team science experience, and industrial nouse. I felt that there was a need for a hybrid construct. Being driven by a combination of intense curiosity about cancer science and an equally strong desire to make a difference for patients with cancer (not least since my father had died from colorectal cancer), I was attracted by the opportunity offered by ICR to build a large drug discovery team in academia that combined the respective strengths of academic and industrial research. There was already a small drug discovery group at ICR that had successfully discovered clinical cancer drugs, mainly chemotherapy agents, and there was support for me to continue that culture while also modernizing in terms of technologies and focusing on new molecular targets. Over time, we grew the Unit to around 160 staff, of which about half were core-funded on a 5-year renewable program grant from CRUK, providing important critical mass, stability, and flexibility to run a portfolio of projects. Also important was the implementation of what is referred to as the ‘centre of disease excellence model’, which combines (i) close collaboration and cutting-edge basic research as source of targets (through ICR, CRUK, and beyond); (ii) equally close interaction with clinical cancer specialists (through ICR and our clinical partner the Royal Marsden Hospital); and (iii) the recruitment of experienced drug discovery scientists from both academia and industry. Importantly, our drug discovery leaders have full faculty status within ICR and are required to publish high-quality papers as well as discover chemical biology probes and drugs.

Critically, since we are not commercially driven, we see our mission as tackling high-risk projects and obtaining preclinical or clinical proof of concept, before partnering with industry at the right time for each project. We put great emphasis on robustness and reproducibility in our thorough target validation using orthogonal methods with both genetic tools and chemical probes. While operating industry-like multidisciplinary project teams with milestone-driven objectives, we at the same time can give promising projects time and resource to succeed rather than killing them too early. We recognize the importance of getting the right people to work together. We have a clear strategy but recognize that getting the right discovery and translational culture is even more important.

Success is shown by our discovery since 2005 of 20 preclinical drug candidates, progress of nine of these into clinical trials, and achieving the regulatory approval of the CYP17 inhibitor abiraterone, discovered at ICR and trialed clinically at the Royal Marsden, for treatment of advanced prostate cancer. We have also won numerous awards, including the AACR Team Science prize in 2012, which I am specially proud of.

Over the next 5 years, the Unit is embracing the tough challenge of overcoming cancer evolution and drug resistance. Probably the biggest challenge we face is finding sufficient funding to bridge the ‘Valley of Death’ between identifying a preclinical candidate drug and progressing it rapidly through to early clinical trials. We are identifying and helping to create various innovative funding mechanisms to bridge the gap. Now that I am Chief Executive of and President of ICR, we have recruited Raj Chopra from Celgene to lead this next phase.

Thinking about academic drug discovery more broadly, I think it is encouraging to see this model developing elsewhere. I believe that many of the above learning points are applicable in other places. I think there is increasing recognition that academic drug discovery is not only about running high-throughput screens, but also requires considerable investment and multidisciplinary team science expertise to go all the way from target discovery through and into the clinic. Collaboration and partnership is essential. Within the new culture in industry of outsourcing innovation, there is increasing potential for academic drug discovery to have a valuable role in the ecosystem.

## What are the priorities over the next decade in cancer drug discovery?

I think it is worth noting that the War on Cancer, as articulated during the 1970s, greatly underestimated the difficulty of treating more than 200 different cancers that have, as we now know, even greater genetic subdivisions. In addition, the expectation of discovering what some perceived would be a single magic bullet cure for all these different cancers in 5 years was of course hopelessly ill-conceived. Medical and scientific challenges are always tough to crack and timescales for breakthroughs are difficult to predict. Despite this, real progress has been made. For example, figures for the UK show that overall cancer survival is now double what it was during the 1970s, with more than half of all patients with cancer surviving 10 years or more. Chemotherapy and molecularly targeted drugs have certainly had an important role in that.

By contrast, survival of many cancers, such as those of the lung, pancreas, gullet, and brain, is still very poor indeed. The 5-year survival for lung cancer in the UK is less than 10%

I think that without doubt the biggest priority both in the clinic and in cancer research is understanding and overcoming the challenges of treatment resistance, which is commonly driven by genetic instability leading to tumor heterogeneity and clonal evolution. At my own Institute, we have positioned this challenge front and central in our new research strategy for 2016-2021ii.

Resistance commonly involves Darwinian selection, but rather than the survival of the fittest, I refer to this as ‘the survival of the nastiest’, which seems more appropriate. The selective pressure for such malign evolution can be provided by the adverse conditions of the tumor microenvironment and also unfortunately by drug therapy. There are a number of ways to tackle this enduring problem. First of all, we need to extend the druggable cancer genome as I described earlier. Second, we need to consider that targeting driver oncogenic kinases and epigenetic regulators may routinely provide only 3–6 months of valuable life extension before resistance develops. This indicates to me that oncogenic drivers should not be the sole focus of our drug discovery efforts and targeting non-oncogene addiction mechanisms could be a valuable part of the mix, as exemplified by proteasome inhibitors for example. Third, we must discover the most effective drug combinations. By using drugs in appropriate cocktails, we can hopefully cut off cancer's evolutionary escape routes. These combinations will need to include enhancing the immune response against the cancer. Indeed, the approval of immune checkpoint inhibitors has been the standout breakthrough of the past few years. Fourth, we need to discover cancer network drugs. Network drugs tackle more than one of the cellular signaling pathways that are hijacked in cancer. These drugs can hit cancer harder than drugs targeted to only one protein, because they can act on several targets at once. One example I am interested in is the use of HSP90 molecular chaperone inhibitors that show promise in clinical trials against drug-resistant cancers, including lung tumors driven by exon 20-mutant EGFR. Our own drug called luminespib that we discovered at ICR in collaboration with Vernalis is showing promising activity in this type of cancer, which is unresponsive to other drugs.

Although we now recognize that drug resistance arising through adaptive mechanisms and clonal evolution will be an enduring problem, I think that our increasing understanding of the mechanisms underlying resistance will allow us to focus on the best targets for combinatorial intervention and the best clinical strategies to apply to stay ahead of the game. If we do this, I am confident that we should make major improvements in long-term survival and cure rates for cancer.

## Resources

^i^ www.icr.ac.uk/blogs/the-drug-discoverer/page-details/my-reflections-on-the-death-of-cancer)

^ii^ http://d1ijoxngr27nfi.cloudfront.net/default-document-library/icr-royal-marsden-research-strategy-2016-2021.pdf?sfvrsn=6

